# Perspectives on decision making amongst older people with end‐stage renal disease and caregivers in Singapore: A qualitative study

**DOI:** 10.1111/hex.12943

**Published:** 2019-08-16

**Authors:** Emeline Han, Victoria Haldane, Joel Jun Kai Koh, Rina Yu Chin Quek, Semra Ozdemir, Eric Andrew Finkelstein, Tazeen Hasan Jafar, Hui‐Lin Choong, Sheryl Gan, Lydia W. W. Lim, Farah Shiraz, Helena Legido‐Quigley

**Affiliations:** ^1^ Saw Swee Hock School of Public Health National University of Singapore Singapore Singapore; ^2^ Duke NUS Medical School Singapore Singapore; ^3^ Department of Renal Medicine Singapore General Hospital Singapore Singapore; ^4^ London School of Hygiene and Tropical Medicine London UK

**Keywords:** decision making, dialysis, end‐stage renal disease, haemodialysis, peritoneal dialysis or conservative management, qualitative research

## Abstract

**Background:**

End‐stage renal disease (ESRD) is increasing both globally and in Asia. Singapore has the fifth highest incidence of ESRD worldwide, a trend that is predicted to rise. Older patients with ESRD are faced with a choice of haemodialysis, peritoneal dialysis or conservative management, all of which have their risks and benefits.

**Objective:**

This study seeks to explore perspectives on decision making amongst older (≥70) Singaporean ESRD patients and their caregivers to undergo (or not to undergo) dialysis.

**Design:**

Qualitative study design using semi‐structured interviews.

**Setting and participants:**

Twenty‐three participants were recruited from the largest tertiary hospital in Singapore: seven peritoneal dialysis patients, five haemodialysis patients, four patients on conservative management and seven caregivers.

**Results:**

While some patients believed that they had made an independent treatment decision, others reported feeling like they had no choice in the matter or that they were strongly persuaded by their doctors and/or family members to undergo dialysis. Patients reported decision‐making factors including loss of autonomy in daily life, financial burden (on themselves or on their families), caregiving burden, alternative medicine, symptoms and disease progression. Caregivers also reported concerns about financial and caregiving burden.

**Discussion and conclusion:**

This study has identified several factors that should be considered in the design and implementation of decision aids to help older ESRD patients in Singapore make informed treatment decisions, including patients' and caregivers' decision‐making factors as well as the relational dynamics between patients, caregivers and doctors.

## 1 INTRODUCTION

With trends showing increasing incidence, end‐stage renal disease (ESRD) is a growing public health concern globally.^1^ In Singapore, a small, densely populated and rapidly ageing city‐state, ESRD incidence has almost doubled between 1999 and 2015,^2^ making it the fifth highest incidence worldwide.^3^ These figures are expected to continue rising due to population ageing and a high local prevalence of diabetes.^4^

Haemodialysis (HD) and peritoneal dialysis (PD) are primary therapies for ESRD, and evidence from Singapore suggests that for younger and healthier ESRD patients, PD is the most cost‐effective treatment choice.^5^ However, there is increasing evidence of the lack of survival advantage of dialysis over conservative management (CM), which focuses on symptom management, for ESRD patients over the age of 75 and with multiple comorbidities.^6^, ^7^ Given that there is no clear optimal treatment strategy for older ESRD patients, many factors may influence their decision on which treatment to pursue. These include average life expectancy, quality of life, caregiver burden, availability of transport and cost.^8^, ^9^

As ESRD patients and their caregivers navigate these choices, there is a need for supportive, contextually appropriate decision‐making aids to enable informed decision making. Evidence from Singapore has shown that lack of knowledge of CM and deference to physician recommendations may lead older ESRD patients to seek dialysis^10^ and that a small proportion of patients later regret their decision to start dialysis.^11^ Work done elsewhere has also shown that most older patients have unrealistic expectations of dialysis upon commencing treatment,^12^ and has suggested the need for age‐specific information tailored to older ESRD patients that clearly communicate the risks, benefits and burdens associated with dialysis.^13^

Presently, there is limited research exploring the treatment decision‐making process of older ESRD patients and caregivers, and the majority of current literature is centred on Western populations. As rates of ESRD and associated risk factors rise in Asian populations, it is important to better understand the decision‐making process of patients and caregivers within Asian contexts. Singapore's culture and policy structures are uniquely and deeply rooted in the principles of self‐reliance and family as the first line of support.^14^, ^15^ Individual and family responsibility are emphasized in health‐care policies such as Medisave, a mandatory saving scheme that requires citizens to set aside a portion of their income to pay for their own or immediate family members' medical expenses.^16^ This paper thus seeks to explore perspectives on decision making amongst older Singaporean ESRD patients and their caregivers to inform the development and implementation of contextualized and culturally appropriate decision‐making aids.

## 2 MATERIALS AND METHODS

This qualitative study followed the guidelines of the Consolidated Criteria for Reporting Qualitative Studies (Appendix S2).

### 2.1 Setting

The study was conducted at Singapore General Hospital, the largest tertiary hospital in Singapore.

### 2.2 Participants and recruitment

This study used two methods to sample key informants: purposive sampling to recruit patients with ESRD and snowball sampling to recruit their caregivers. The inclusion criteria were as follows: patients had incident chronic kidney disease Stage 5 (glomerular filtration rate < 10 mL/min), were aged 70 years or older and were currently receiving HD, PD or CM. Caregivers were primary informal caregivers who had a close personal relationship with the patient and were aged 21 years or older.

**Figure 1:**
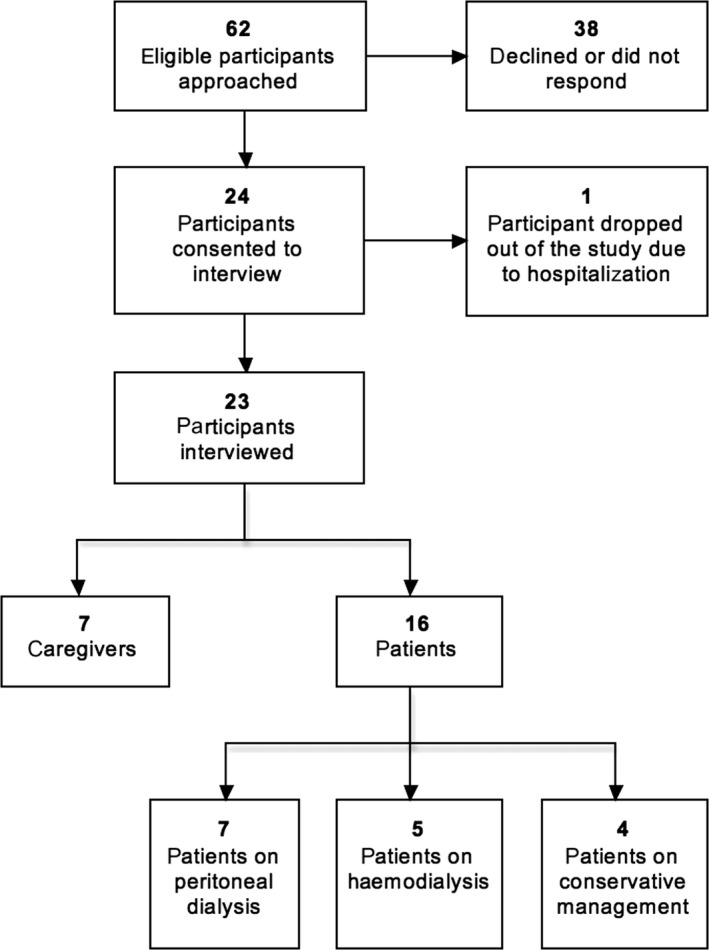
Participant recruitment flow chart

Participants were approached and recruited face‐to‐face by researchers (EH, JK, VH, RQ) during appointments at Singapore General Hospital with the assistance of renal coordinators, nurses and doctors. In total, 62 participants were approached; 38 declined or did not respond, and one dropped out due to hospitalization. Reasons for refusal included being too tired and frail to have a prolonged conversation or not wanting to talk about their experience. Twenty‐three participants from four participant categories were included in the study: seven PD patients, five HD patients, four patients on CM and seven caregivers. Of these participants, 14 were female and nine were male. All but three participants were Chinese. Patients had a mean average age of 75 (range 71‐85). Figure 1 displays a flow chart of the participant recruitment process, and Table 1 shows the basic demographic characteristics of each included participant.

**Table 1:** Participant characteristics table

ID	Pseudonym	Gender	Age range	Ethnicity	Treatment type
Patient characteristics					
PA08	Bee Eng	F	76‐80	Chinese	CM
PA12	Irene	F	71‐75	Chinese	CM
PA19	Chiang Tee	M	71‐75	Chinese	CM
PA31	Wen Xi	M	71‐75	Chinese	CM
PB09	Ai Jia	F	76‐80	Chinese	HD
PB11	Siew Leng	F	76‐80	Chinese	HD
PB24	Mei Leng	F	71‐76	Chinese	HD
PB31	Huang	M	76‐80	Chinese	HD
PB36	Vivian	F	76‐80	Chinese	HD
PC05	Seng	M	71‐75	Chinese	PD
PC10	Nur	F	81‐85	Malay	PD
PC11	Leong	M	71‐75	Chinese	PD
PC16	Larry	M	71‐75	Chinese	PD
PC32	Priya	F	71‐75	Indian	PD
PC35	Fang	F	76‐80	Chinese	PD
PC42	Chia	M	71‐75	Chinese	PD
Caregiver characteristics					
CPA08	Chin Boon	M	Spouse	Chinese	CM
CPA25	Cynthia	F	Child	Malay	PD
CPB24	Jun Hao	M	Child	Chinese	HD
C1PB31	Alice	F	Godchild	Chinese	HD
C2PB31	Patricia	F	Godchild	Chinese	HD
CPC42	Ying	F	Spouse	Chinese	PD
CPC44	Eng	F	Spouse	Chinese	PD

### 2.3 Data collection

Semi‐structured, in‐depth interviews were conducted with all 23 participants by researchers (JK, EH, RQ, VH, HLQ) trained in the study protocol, interview guide and qualitative research methods. Interviews were conducted in English (n = 8), Chinese (n = 9) or Malay (n = 1) by researchers fluent in that language. All caregivers were interviewed jointly with patients except two who were interviewed alone as the patient declined to participate in the study. Efforts were made to ensure that both the caregiver and patient had the opportunity to contribute equally in these dyadic interviews,^17^ such as probing for the other participant's opinions if only one of them responded initially.

Interviewers followed an interview guide (Appendix S1) while conducting interviews. The guide included topics such as sociodemographics and caregiver support, medical history and medications, doctor‐patient relationships, decision‐making processes and lived experiences. Interviews primarily took place in participants' homes or waiting areas within the hospital (at the request of participants). Each interview lasted approximately 30‐60 minutes. Audio recordings and field notes were taken during all the interviews. Data collection ceased when the researchers were confident that data saturation had been reached, which is defined by Saunders et al^18^ as the point at which no new information is being generated by additional interviews. 

### 2.4 Ethics

Informed consent for participation and recording was obtained before the interviews started, and participants signed a Participant Information Sheet and Consent Form in their language of preference (Chinese, Malay or English). Participants could refuse to answer any of the questions and/or discontinue their participation in the research at any time. Efforts were made to conduct interviews in private, quiet places that individual participants found comfortable and appropriate. All interview materials were also stored securely to ensure data confidentiality. Each excerpt in this paper includes the number of the interview and code letters (F for female, M for male) so that extracts from the same individual can be linked. To maintain anonymity, all names reported are pseudonyms and identifying data have been excluded.

### 2.5 Reliability and validity

In establishing the rigour of our enquiry, we employ the four trustworthiness criteria for qualitative research developed by Lincoln and Guba: credibility, dependability, confirmability and transferability.^19^ To increase the credibility and dependability of our research, investigator triangulation and stepwise replication were implemented. This involved multiple investigators analysing the same data set separately, comparing their interpretations of the data and resolving inconsistencies. Where interpretations differed, the investigators discussed until consensus was reached on the interpretation that was most consistent with the original meaning of the data. Confirmability of the findings was also secured by requiring all investigators to keep reflexive notes and participate in regular meetings to ensure that interpretations of the findings were not influenced by personal opinions and biases but were grounded in the data. Finally, the transferability of our study has been facilitated by the use of purposive sampling and detailed descriptions of the study methodology and context.

### 2.6 Patient and public involvement (PPI)

Patient and public involvement in research is not a common practice in Singapore for a variety of reasons, including lack of funding. Consequently, patients were not involved in the design and conduct of the study, interpretation of results or the writing of this paper.

### 2.7 Data analysis

Interviews were recorded and then translated into English (if necessary) and transcribed in full. To ensure reliability, professional bilingual transcriptionists who were familiar with local culture and colloquialisms were hired to translate and transcribe the interviews simultaneously. The respective interviewers (EH, JK, RQ) then double‐checked all transcripts against the audio recordings and rectified any missing or misinterpreted information to ensure semantic and conceptual accuracy. This approach was preferred to back and forward translation, which is usually insufficient to obtain equivalence in meaning.^20^ Three researchers (EH, JK and VH) coded interviews using QSR Nvivo 11 Software utilizing a combination of inductive and deductive thematic analysis. A priori themes were first derived from the research aims, interview questions and previous literature. Next, a posteriori themes were generated from the data and refined using techniques from grounded theory, including the constant comparative method and line‐by‐line analysis to test preliminary assumptions.^21^ Data analysis ceased when the researchers agreed that inductive thematic saturation had been achieved, which is defined by Saunders et al as the point at which no new codes or themes are emerging from the data. Triangulation was carried out regularly throughout the analysis process by comparing independently coded data sets and discussing disagreements until consensus was reached on the final list of codes and themes.^18^

### 2.8 Conceptual framework

**Figure 2:**
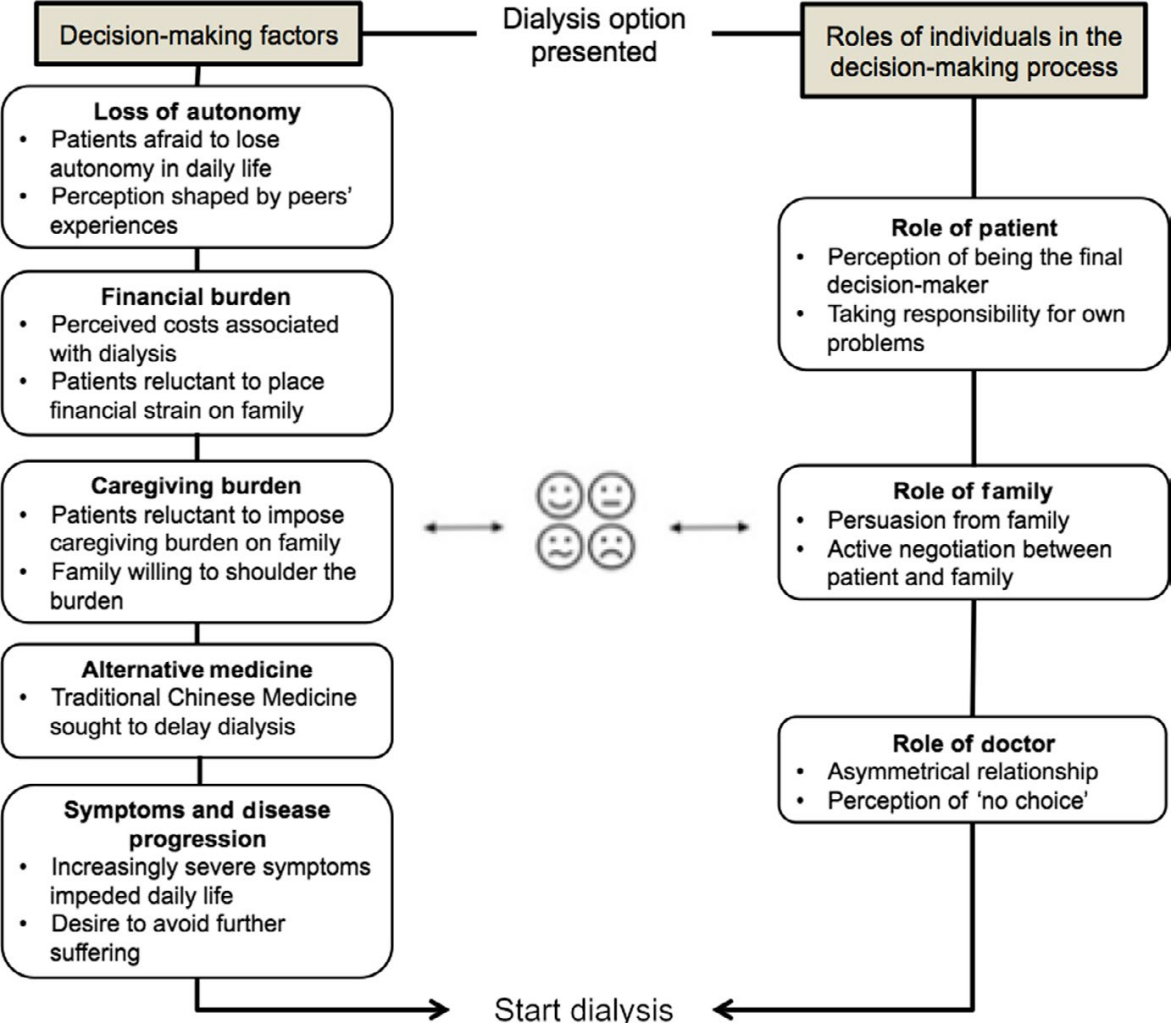
Conceptual framework of decision making for ESRD patients

We used a conceptual framework to guide our analysis of patient and caregiver treatment decision making (Figure 2). We first considered the role of socio‐economic factors, as well as doctor‐patient and caregiver‐patient relationships, in the decision‐making processes of older ESRD patients and their families.^22^, ^23^, ^24^ We then used emergent themes to better inform the structure and content of our framework.

## 3 RESULTS

Table 2 presents a summary of the key themes, subthemes and examples of evidence. Firstly, we will describe the roles of patients, families and doctors in the decision‐making process. Secondly, we will explore the factors that informed patients' and families' initial decision to accept or decline dialysis, as well as factors that caused patients who initially declined dialysis to start it. All quotes by patients are presented with the accompanying ID number, pseudonym, gender, age range, ethnicity and treatment type (HD/PD/CM). Quotes by caregivers are presented with their ID number (matched with the patient's), pseudonym, gender, relationship to the patient and the patient's treatment type.

**Table 2:** Key themes and examples of evidence

Theme	Subtheme	Examples of evidence
Role of patient in decision making	Perception of an independent decision	‘Interviewer: So you all made a decision together? Patient: No. I made the final decision because I'm the person in trouble, they support’. [PC11_Leong_M_ 71‐75_Chinese_PD]
Role of family in decision making	Persuasion by family	‘He initially refused to undergo dialysis and delayed it for a long time because he didn't want to pose a burden on our children. He only underwent it after our children persuaded him… He only gave in after our children cried and kneeled down to beg him’. [CPC42_Ying_F_PD_Spouse]
Role of doctor in decision making	Perception of ‘no choice’	‘Patient: So I told the doctor and the doctor told me that I had to undergo dialysis. Caregiver: There was no choice; we had to start dialysis straightaway’. [PB31_Huang_M_ 76‐80_Chinese_HD] [C1PB31_Alice_F_HD_Godchild]
	Advice by doctor	‘He would use positive words to help us and advise us. It's because of him that I accepted dialysis. Otherwise, I wouldn't have accepted it’. [PC42_Chia_M_ 71‐75_Chinese_PD]
Decision‐making factors	Loss of autonomy in daily life	‘But I've seen many people here in wheelchairs who keep drooling and need people to feed them; to be honest it's better to die than to lead such a life. What is the point of prolonging such a life? Therefore I said that I don't want to undergo dialysis’. [PA19_Chiang Tee_M_ 71‐ 75_Chinese_CM]
	Financial burden	‘I mean you got children but you don't want to be a burden to them also, for nothing you know dialysis is running thousands of dollars for what. I mean inside me I really regret it's a burden to my daughter but naturally, she won't accept that. She says that's a duty of a daughter to her mother you know according to her’. [PC32_Priya_F_71‐75_Indian_PD]
	Caregiving burden	‘Even if your son is capable, he has to take care of himself and his own family. Does he still have to take care of you? Can you bear to ask him to take care of you? If he starts taking care of you, he cannot stop taking care of you; but if he continues taking care of you, it's a problem too’. [PA19_Chiang Tee_M_71‐75_Chinese_CM]
	Alternative medicine	‘The [TCM] doctor there said that, if there's need for dialysis, at last, you also must go. Not to say that the Chinese herbs will cure you, will help you to improve anything… just to control it, that's all*…*not enough*…*doctor does say that’. [PB36_Vivian_F_76‐80_Chinese_HD]
	Symptoms and disease progression	‘He only came for dialysis because it reached the point where he couldn't breathe properly… By then, he had no choice as he was suffering… He told us to just let him die. But the doctor told him that he might not die right away and instead suffer in a vegetative state’. [CPC42_Ying_F_PD_Spouse]

### 3.1 Roles of individuals involved in the decision‐making process

Some patients reported that they had made an independent treatment decision, while others reported feeling like they had no choice in the matter or that they were strongly persuaded by their doctors and/or family members to undergo dialysis.

#### 3.1.1 Role of patient

Many patients stressed that the final decision to accept or reject dialysis was theirs to make and that they made it on their own, even if they had consulted with health‐care professionals and family members during the decision‐making process:

Interviewer: So you all made a decision together?

Patient: No. I made the final decision because I’m the person in trouble, they support. [PC11_Leong_M_71‐75_Chinese_PD]

This insistence on making an independent decision largely stemmed from a notion of self‐reliance and taking personal responsibility for one's own problems. Patients expressed a reluctance to implicate others in the consequences of their treatment decision:

Interviewer: Back then, when you were asked to undergo dialysis, did you discuss it with your children?

Patient: Yes, but I was told to make the decision myself. This decision cannot be made by others.

Interviewer: Please explain more.

Patient: If they make the decision for me to undergo dialysis, it means they will have to take care of me. [PA19_Chiang Tee_M_71‐75_Chinese_CM]

#### 3.1.2 Role of family

Families played a substantial role in the treatment decision‐making process, often by persuading patients to start dialysis. Notably, several patients in our study acceded to the wishes of their children for them to undergo dialysis.

The wife of a PD patient recounted how her husband eventually acquiesced to dialysis after receiving very strong and persistent persuasion from their children:

He initially refused to undergo dialysis and delayed it for a long time because he didn't want to pose a burden on our children. He only underwent it after our children persuaded him… He only gave in after our children cried and kneeled down to beg him. [CPC42_Ying_F_PD_Spouse]

These narratives reflected a process of negotiation between patients and families and showed the powerful influence that families had over patients' decisions.

#### 3.1.3 Role of doctor

The relationship that patients have with their doctors is complex and has a bearing on their treatment decision‐making process. Many patients in this study had an asymmetrical relationship with their doctor, in which the doctor's opinions held a lot more weight than their own. These patients felt that they were not in a position to question their doctors' recommendations because doctors were more educated than them about medical affairs:

I also don't know what is dialysis…how do I know, those days old people where got school…we know nothing…whatever they say we just listen only…the one who listen just the children so they would know….I just heard people said only…but who are we to say anything? We are not doctors. [PC10_Nur_F_81‐85_Malay_PD]

Based on the foundations of such an asymmetrical relationship, many patients and caregivers felt like they did not have a choice but to undergo dialysis when their doctors presented it as a necessity:

Patient: So I told the doctor and the doctor told me that I had to undergo dialysis.

Caregiver: There was no choice; we had to start dialysis straightaway. [PB31_Huang_M_76‐80_Chinese_HD] [C1PB31_Alice_F_HD_Godchild]

On the other hand, some patients described accepting the advice of their doctors to undergo dialysis within a positive doctor‐patient relationship as doctors made an effort to encourage, reassure and support them during the decision‐making process:

He would use positive words to help us and advise us. It's because of him that I accepted dialysis. Otherwise, I wouldn't have accepted it. [PC42_Chia_M_71‐75_Chinese_PD]

### 3.2 Decision‐making factors

Common factors that patients considered when making the decision to undergo dialysis included loss of autonomy in daily life, financial burden (on themselves or on their families), caregiving burden, alternative medicine, symptoms and disease progression. Caregivers also reported concerns about financial and caregiving burden.

#### 3.2.1 Loss of autonomy in daily life

Many patients were afraid that they would lose autonomy in their daily life if they commenced dialysis and this was a crucial factor in their decision making. For example, Vivian, who originally rejected dialysis, thought that it was meaningless to prolong her life with dialysis because she believed that the time‐consuming nature of dialysis and its side‐effects would restrict her daily life and take away her freedom to travel:

I said, “I'm not in for anything why I extend my life?” Extend this type of life is no good. You bear for the pain, you got to take medicine every day, go for dialysis…no doubt one week three times…the other three days, gone. You are tired, you cannot go anywhere, even Johor Bahru also cannot go. [PB36_Vivian_F_76‐80_Chinese_HD]

For many patients, these perceptions were shaped by witnessing peers undergo dialysis and the negative impacts that it had on their lives. For example, Chiang Tee, who has so far chosen not to start dialysis, described how he saw his friend become wheelchair‐bound and dependent on a helper for his daily activities, and how he perceives such a life to be worse than death:

I've seen many people here in wheelchairs who keep drooling and need people to feed them; to be honest it's better to die than to lead such a life. What is the point of prolonging such a life? Therefore, I said that I don't want to undergo dialysis. [PA19_Chiang Tee_M_71‐75_Chinese_CM]

The quotes reveal that for these patients, it was quality rather than quantity of life that featured as a leading consideration when making their treatment decision.

#### 3.2.2 Financial burden

The financial burden of dialysis was raised as a significant decision‐making factor by many patients, who perceived that treatment expenses would be extremely burdensome for themselves or their families.

For example, Irene, who is currently still deciding whether to start dialysis, shared that the cost of dialysis is her primary consideration. As she does not have family support, she is worried about being able to afford dialysis without incurring debt:

I cannot be spending above my budget. I don't want to be in debt. Ah, that is the number one thing… Because I don't have family support…I don't want to depend on anybody…And I also don't want to be obligated to my church members. [PA12_Irene_F_71‐75_Chinese_CM]

On the other hand, Priya related how she initially refused to undergo dialysis because she was reluctant to place a financial strain on her daughter, but her daughter regarded it as her ‘duty’ to provide financial support for her mother's care:

I mean you got children but you don't want to be a burden to them also, for nothing you know dialysis is running thousands of dollars for what. I mean inside me I really regret it's a burden to my daughter but naturally, she won't accept that. She says that's a duty of a daughter to her mother you know according to her. [PC32_Priya_F_71‐75_Indian_PD]

In short, while many patients were hesitant to start dialysis because of the perceived financial burden, the family caregivers in our study did not regard the cost of dialysis as a reason to reject it but rather as a challenge they should and could find ways to cope with.

#### 3.2.3 Caregiving burden

Patients recognized that their decision to undergo dialysis would give rise not just to financial but also caregiving obligations for their family, and this was a major factor in their decision making.

For example, one of the main reasons why Chiang Tee rejected dialysis was because he could not bear to pose a long‐term caregiving burden on his son:

Even if your son is capable, he has to take care of himself and his own family. Does he still have to take care of you? Can you bear to ask him to take care of you? If he starts taking care of you, he cannot stop taking care of you; but if he continues taking care of you, it's a problem too. [PA19_Chiang Tee_M_71‐75_Chinese_CM]

While patients' families also acknowledged the caregiving responsibilities of dialysis as a genuine concern, it did not deter them from encouraging patients to undergo dialysis:

Our children told him that they are willing to shoulder the burden and he needs to take care of himself. After much persuasion, he finally conceded. Before that, he totally refused. He even refused when the doctor told him to undergo dialysis… [CPC42_Ying_F_PD_Spouse]

In short, while the caregiving burden associated with dialysis was a prominent reason for patients to reject it, the families in our study showed a willingness to shoulder this burden and even persuaded patients to undergo dialysis.

#### 3.2.4 Alternative medicine

Furthermore, some patients stated that they preferred to try alternative medicine, in particular traditional Chinese medicine (TCM), before accepting dialysis. Only when TCM was perceived to no longer be an effective mode of treatment because of the severity of their conditions did these patients resort to dialysis.

In particular, Vivian understood that TCM would not provide a cure for her kidney disease, but she still sought TCM to delay the initiation of dialysis:

The [TCM] doctor there said that, if there's need for dialysis, at last, you also must go. Not to say that the Chinese herbs will cure you, will help you to improve anything… just to control it, that's all…not enough…doctor does say that. [PB36_Vivian_F_76‐80_Chinese_HD]

On the other hand, Chiang Tee claims that he has been cured by a folk remedy that was recommended to him by a friend and available on the Internet, which is why he believes that he does not need to undergo dialysis anymore:

After that, someone introduced to me to a concoction of lychee seeds and pig kidney. 7 lychee seeds and pig kidneys. You know, when one is not afraid of death, everything goes. So I tried drinking it. My friend's wife took care of me by boiling that soup for me. I drank that three times and SGH told me to take a blood test. After the blood test, the female doctor told me that I don't have to visit the doctor for kidney problems anymore. I asked, am I supposed to just wait for death? She said no, it's good; I don't have to undergo dialysis. [PA19_Chiang Tee_M_71‐75_Chinese_CM]

#### 3.2.5 Symptoms and disease progression

Finally, many patients explained how their decision to initiate dialysis was strongly influenced by increasingly severe symptoms that occurred alongside disease progression. This was a determining factor for several patients who initially declined but subsequently accepted dialysis.

For example, Siew Leng described how she originally rejected dialysis because she was young and symptom‐free, but extreme swelling in her body eventually drove her to initiate dialysis because she could no longer walk:

At that time I was young, what for I go for dialysis? Nothing wrong with me. I'm okay, I can walk, and I can do anything. After that I just forget about it, nothing wrong…until you know what happened? I was all swollen, my leg, my hand, even my feet, all swollen with water. What to do? So swollen I can't walk, so have to go for dialysis, that's how I started. [PB11_Siew Leng_F_76−80_Chinese_HD]

Ying also conveyed that it was only when the patient began to experience breathing difficulties that he decided to commence dialysis, not out of a desire to prolong his life but to avoid further suffering:

He only came for dialysis because it reached the point where he couldn't breathe properly… By then, he had no choice as he was suffering… He told us to just let him die. But the doctor told him that he might not die right away and instead suffer in a vegetative state. [CPC42_Ying_F_PD_Spouse]

In other words, many patients only decided to start dialysis when their symptoms were beginning to affect their everyday functioning and quality of life, which is consistent with their concerns regarding loss of autonomy in their daily life.

## 4 DISCUSSION

This qualitative study explored perspectives and experiences of the decision to undergo dialysis amongst older ESRD patients and their caregivers in Singapore ahead of a study to design ESRD patient decision‐making aids. We organized our findings based on a framework to understand the patient decision‐making journey and the various factors that influence this decision. Based on our findings, we make some recommendations regarding the creation and implementation of decision‐making guides.

### 4.1 Key findings and recommendations

Patients' decision‐making process involved weighing the perceived benefits and risks of dialysis, which evolved over time and were shaped by personal values on life and death. Some patients explicitly valued quality of life over longevity; rather than fearing death, they feared a life of suffering and dependence on others. These fears were often perpetuated by witnessing peers' negative experiences of dialysis. Hence, for many patients who had not yet suffered severe symptoms of ESRD at the point of decision making, factors such as loss of autonomy in managing daily life, financial burden and caregiving burden tilted the scales against dialysis. Conversely, patients' experience of gradually worsening symptoms that impeded their daily life shifted the balance in favour of dialysis. These results correspond with studies in other countries as well as a qualitative study in Singapore which found that major factors driving patients to decline dialysis included age and life completion, financial and physical costs of dialysis, and the perceived pain and suffering caused by dialysis.^25^, ^26^, ^27^, ^28^, ^29^ Our study adds that many older ESRD patients in Singapore seek alternative medicine such as traditional Chinese medicine (TCM) as a means to delay dialysis, and the main reason why many eventually decide to start dialysis despite initial rejection is to relieve intensifying symptom burden (rather than to extend their lives). Our study also adds that although family members have worries about the caregiving and financial responsibilities associated with dialysis, they demonstrate a willingness to undertake it and often persuade patients to undergo dialysis, emulating the traditional Asian value of filial piety.^30^

Given the above, decision‐making aids for older Singaporean ESRD patients and caregivers should firstly offer an opportunity for dialogue about quality of life after commencing treatment, focusing not just on survival but also on alleviating the impact of dialysis on patients' and their families' daily lives, including how to maintain autonomy in everyday activities (for patients) and handle the practicalities of caregiving (for caregivers). Secondly, many patients and caregivers in our study were unaware of the financial structures available to help them mitigate the cost of dialysis. Decision‐making aids should offer information about access to financial and psychosocial supports that can break down barriers in decision making by defraying the costs associated with dialysis uptake. Thirdly, none of our participants described CM as an option, as was the case in a similar study in the United States.^31^ Patients either saw themselves as rejecting dialysis or regarded CM as an intermediate step before commencing dialysis—none perceived themselves to be actively choosing CM. Given that many patients' primary motivation for starting dialysis was symptom management, decision‐making aids should educate patients on CM, a less costly option that focuses on minimizing symptom burden and maximizing quality of life.^32^ It is imperative that decision aids make patients aware of CM as an option, as well as provide complete information on the relative risks and benefits of CM, HD and PD. Fourthly, as many older ESRD patients in Singapore may first turn to TCM with the understanding that they will undergo dialysis if/when their condition reaches a critical stage, decision‐making aids should clarify any misconceptions patients have about TCM from an evidence‐based perspective and explain renal disease progression alongside any repercussions of late dialysis.

Several patients in our study reported that they had no choice but to comply with their doctors' recommendation to undergo dialysis because of the pervasive assumption that ‘doctors know best’. Their narratives reflected an asymmetrical doctor‐patient relationship in which patients held doctors in high regard and felt powerless to negotiate their care. This type of asymmetrical relationship has been widely reported in care for chronic conditions.^33^, ^34^, ^35^, ^36^ In terms of dialysis decision making, work by Ladin et al^31^ reported similar results, where patients perceived a lack of choice because of the belief that the decision belonged to their physicians or that dialysis was necessary to avoid imminent death. However, unlike patients in that study who perceived doctors' persuasion negatively, some patients in our study perceived doctors' efforts to encourage and advise them as a positive factor that convinced them to start dialysis. Patients in our study also often regarded themselves as the final decision‐maker, even if their decision was to follow the advice of their family members who in turn trusted their doctors' recommendations. Patients' emphasis on making an independent decision was largely driven by a sense of individual responsibility and reluctance to burden the family, reflecting cultural values in Singapore that promote self‐reliance and prioritizing the interests of the family group over that of the self in isolation.^15^, ^29^ These values can be tied back to the Confucian conception of personhood, which comprises both the vertical dimension of a self‐determined, self‐reliant person and the horizontal dimension of a relational, altruistic person.^30^, ^37^ The Confucian view of autonomy recognizes that individuals do not make decisions in a vacuum but within a network of relations with others.

This complex dynamic between ESRD patients, caregivers and doctors highlights the need for contextualized and culturally relevant decision‐making aids that meets the needs of all parties involved in the decision‐making process.^38^ Decision aids can equip patients and caregivers with greater knowledge, reduce decisional conflict due to feeling uninformed or unclear about personal values, and empower them to participate more actively in decision making.^39^ However, decision aids may not be able to overcome the strong influence of physician recommendations. A recent survey in Singapore revealed that if patients were educated on CM as an option, 54% of patients and 42% of caregivers would choose CM, but if their physician recommended dialysis, 49% of patients and 68% of caregivers would then change their decision.^10^ Therefore, decision aids for ESRD patients and caregivers should be implemented in tandem with decision counselling by different professionals (i.e. not just physicians but also nurses, medical social workers etc) who are trained in shared decision making, bias awareness and communication skills to support patients in making decisions that are consistent with their values and preferences.^40^, ^41^ In order to respect patients' autonomy in the fuller, relational sense, professionals must also learn how to involve families in decision making while protecting patients from undue pressure.^42^ This necessitates training on resolving patient‐family disagreements and treading the fine line between persuasion and coercion, the latter of which has been defined as the use of ‘threats, harassment, berating, intimidation, or other manipulative tactics designed to force vulnerable patients to change well‐established beliefs or preferences’.^43^ Another article examining end‐of‐life decision making in Singapore has similarly advocated for a multidisciplinary team approach that provides more holistic and objective support for decision making to counter the dangers of pure physician‐led paternalism or family‐led determination.^44^

### 4.2 Strengths and limitations

This study is the first study to focus on the perspectives and experiences of ESRD patients above the age of 70 in an Asian population. It is also the first study to include the views of family caregivers to capture the complex inter‐relational dynamics in decision making for this population. A further strength of our study was the inclusion of participants from multiple ethnic backgrounds (Chinese, Malay and Indian), with interviews conducted in the language of participants' preference, which adds to the diversity of experiences and factors reported.

Nonetheless, our sample was still largely dominated by Chinese Singaporeans, who make up more than 75% of Singapore's total population.^45^ Another limitation of the study is the underrepresentation of certain patient groups including those taking conservative management for ESRD from community clinics. Our patient population was also comprised of older patients, who have been found to report higher levels of satisfaction with care than younger groups.^46^ Furthermore, many patients approached had declined to be interviewed, due to either being too frail to hold a long conversation or not wanting to talk about their treatment choice. This could have skewed the views reported in this study, which reflects the perspectives of patients who were more open to talking about their decision‐making experience. Lastly, there was a lack of PPI in this study, as the practice is not commonly adopted in Singapore. Given that our study sought to understand patient decision making and inform patient decision‐making aids, PPI could have helped to improve the relevance of our research to patients, strengthen the validity of our findings and recommendations, as well as increase the usefulness of our research outputs. The potential of introducing patient and public involvement to research in Singapore thus merits serious consideration.

## 5 CONCLUSION

This study has identified several factors from patient and caregiver perspectives to consider in the creation and implementation of decision‐making aids for older ESRD patients in Singapore. These include patients' and caregivers' treatment decision‐making factors (loss of autonomy in daily life, financial burden, caregiving burden, alternative medicine, symptoms and disease progression), as well as the relational dynamics between patients, caregivers and doctors. These factors should be taken into account in the development of decision‐making aids to help older patients make informed and autonomous treatment decisions. In addition, decision‐making aids should be implemented in tandem with decision counselling by a multidisciplinary team of professionals who are trained in shared decision making, including how to involve families in the decision‐making process while protecting patients from undue family pressure or medical paternalism. Future research in this field should explore in greater detail the experiences of Malay and Indian patients and their caregivers, as well as consider the inclusion of PPI to strengthen the design of such work.

## CONFLICT OF INTEREST

None declared.

## AUTHORS' CONTRIBUTIONS

Emeline Han and Joel Jun Kai Koh collected the data, analysed the data, and drafted and revised the manuscript. Victoria Haldane conceived and designed the study, collected the data, analysed the data and drafted the manuscript. Rina Yu Chin Quek collected the data and revised the manuscript. Semra Ozdemir, Eric Andrew Finkelstein, Hui‐Lin Choong, Sheryl Gan, Lydia WW Lim and Tazeen Jafar conceived and designed the study and revised the manuscript. Farah Shiraz revised the manuscript. Helena Legido‐Quigley conceived and designed the study, collected the data and revised the manuscript.

## DATA AVAILABILITY STATEMENT

Participant consent and ethical approval have not been obtained for data sharing. Due to the confidential and sensitive nature of our data, there are no data that can be disclosed beyond that contained within this paper.

## References

[1] Wetmore J , Collins A . Global challenges posed by the growth of end‐stage renal disease. Renal Replacement Therapy. 2016;2(1):15.

[2] Singapore Renal Registry . Singapore Renal Registry Annual Report 2016. Available at https://www.nrdo.gov.sg/docs/librariesprovider3/default-document-library/singapore-renal-registry-annual-report-2016_1999-till-2016_v5_online_final.pdf?sfvrsn=0. Accessed July 9, 2018.

[3] United States Renal Data System (USRDS) . 2018 Annual data report. Volume 2: end‐stage renal disease in the United States. Chapter 11: international comparisons. 2018. Available at https://www.usrds.org/2018/view/v2_11.aspx. Accessed January 9, 2019.

[4] Tan C , Chan C , Ho C , Wong K , Lee E , Woo K . Health economics of renal replacement therapy: perspectives from Singapore. Kidney Int. 2005;67:S19‐S22. 10.1111/j.1523-1755.2005.09405.x 15752233

[5] Yang F , Lau T , Luo N . Cost‐effectiveness of haemodialysis and peritoneal dialysis for patients with end‐stage renal disease in Singapore. Nephrology. 2016;21(8):669‐677. 26566750 10.1111/nep.12668

[6] Chandna S , Da Silva‐Gane M , Marshall C , Warwicker P , Greenwood R , Farrington K . Survival of elderly patients with stage 5 CKD: comparison of conservative management and renal replacement therapy. Nephrol Dial Transplant. 2010;26(5):1608‐1614. 21098012 10.1093/ndt/gfq630 PMC3084441

[7] Murtagh F , Marsh J , Donohoe P , Ekbal N , Sheerin N , Harris F . Dialysis or not? A comparative survival study of patients over 75 years with chronic kidney disease stage 5. Nephrol Dial Transplant. 2007;22(7):1955‐1962. 17412702 10.1093/ndt/gfm153

[8] Morton RL , Snelling P , Webster AC , et al. Factors influencing patient choice of dialysis versus conservative care to treat end‐stage kidney disease. Can Med Assoc J. 2012;184(5):E277‐E283. 22311947 10.1503/cmaj.111355 PMC3307582

[9] Murray MA , Brunier G , Chung JO , et al. A systematic review of factors influencing decision‐making in adults living with chronic kidney disease. Patient Educ Couns. 2009;76(2):149‐158. 19324509 10.1016/j.pec.2008.12.010

[10] Finkelstein E , Ozdemir S , Malhotra C , et al. Understanding factors that influence the demand for dialysis among elderly in a multi‐ethnic Asian society. Health Policy. 2018;122(8):915‐921. 30007521 10.1016/j.healthpol.2018.06.008

[11] Tan E , Teo I , Finkelstein E , Chan C . Determinants of regret in elderly dialysis patients. Nephrology. 2019;24(6):622‐629. 29736929 10.1111/nep.13400

[12] Stringer S , Baharani J . Why did I start dialysis? A qualitative study on views and expectations from an elderly cohort of patients with end‐stage renal failure starting haemodialysis in the United Kingdom. Int Urol Nephrol. 2011;44(1):295‐300. 21850412 10.1007/s11255-011-0045-4

[13] Schmidt R . Informing our elders about dialysis: is an age‐attuned approach warranted? Clin J Am Soc Nephrol. 2011;7(1):185‐191. 22173860 10.2215/CJN.10401011

[14] Khan H .Social policy in Singapore: a Confucian model?. WBI working paper series. Washington, DC: World Bank; 2001 Available at: http://documents.worldbank.org/curated/en/193101468758956946/pdf/multi0page.pdf. Accessed April 10 2019.

[15] Olayiwola J , Shih J , Shiow S , Wee H . Could values and social structures in Singapore facilitate attainment of patient‐focused, cultural, and linguistic competency standards in a patient‐centered medical home pilot? J Patient Exp. 2015;2(2):37‐42. 28725822 10.1177/2374373515615975 PMC5513634

[16] Ministry of Health (Singapore) . Healthcare schemes & subsidies. 2018 Available at: https://www.moh.gov.sg/cost-financing/healthcare-schemes-subsidies. Accessed April 10, 2019.

[17] Caldwell K . Dyadic interviewing: a technique valuing interdependence in interviews with individuals with intellectual disabilities. Qual Res. 2013;14(4):488‐507.

[18] Saunders B , Sim J , Kingstone T , et al. Saturation in qualitative research: exploring its conceptualization and operationalization. Qual Quant. 2017;52(4):1893‐1907. 29937585 10.1007/s11135-017-0574-8 PMC5993836

[19] Lincoln YS , Guba EG , Pilotta JJ . Naturalistic Inquiry. Newbury Park, CA: Sage; 1985.

[20] Sumathipala A , Murray J . New approach to translating instruments for cross‐cultural research: a combined qualitative and quantitative approach for translation and consensus generation. Int J Methods Psychiatr Res. 2006;9(2):87‐95.

[21] Charmaz K . Constructing Grounded Theory: A Practical Guide Through Qualitative Analysis. London. UK: Sage; 2006.

[22] Davison S . End‐of‐life care preferences and needs: perceptions of patients with chronic kidney disease. Clin J Am Soc Nephrol. 2010;5(2):195‐204. 20089488 10.2215/CJN.05960809 PMC2827591

[23] Morton R , Tong A , Howard K , Snelling P , Webster A . The views of patients and carers in treatment decision making for chronic kidney disease: systematic review and thematic synthesis of qualitative studies. BMJ. 2010;340(jan19 2):c112. 20085970 10.1136/bmj.c112 PMC2808468

[24] Tversky A , Kahneman D . The framing of decisions and the psychology of choice. Science. 1981;211(4481):453‐458. 7455683 10.1126/science.7455683

[25] Axelsson L , Randers I , Lundh Hagelin C , Jacobson S , Klang B . Thoughts on death and dying when living with haemodialysis approaching end of life. J Clin Nurs. 2012;21(15–16):2149‐2159. 22788556 10.1111/j.1365-2702.2012.04156.x

[26] Johnston S , Noble H . Factors influencing patients with stage 5 chronic kidney disease to opt for conservative management: a practitioner research study. J Clin Nurs. 2012;21(9–10):1215‐1222. 22384863 10.1111/j.1365-2702.2011.04001.x

[27] Noble H , Meyer J , Bridges J , Kelly D , Johnson B . Reasons renal patients give for deciding not to dialyze: a prospective qualitative interview study. Dialysis Transplant. 2009;38(3):82‐89.

[28] Visser A , Dijkstra G , Kuiper D , et al. Accepting or declining dialysis: considerations taken into account by elderly patients with end‐stage renal disease. J Nephrol. 2009;22(6):794‐799. 19967659

[29] Seah A , Tan F , Srinivas S , Wu H , Griva K . Opting out of dialysis – exploring patients' decisions to forego dialysis in favour of conservative non‐dialytic management for end‐stage renal disease. Health Expect. 2013;18(5):1018‐1029. 23647805 10.1111/hex.12075 PMC5060897

[30] Ho Z , Radha Krishna L , Yee C . Chinese familial tradition and western influence: a case study in Singapore on decision making at the end of life. J Pain Symptom Manage. 2010;40(6):932‐937. 21145471 10.1016/j.jpainsymman.2010.06.010

[31] Ladin K , Lin N , Hahn E , Zhang G , Koch‐Weser S , Weiner D . Engagement in decision‐making and patient satisfaction: a qualitative study of older patients' perceptions of dialysis initiation and modality decisions. Nephrol Dial Transplant. 2017;32(8):1394-1401. 27576590 10.1093/ndt/gfw307 PMC5837335

[32] Murtagh F , Burns A , Moranne O , Morton R , Naicker S . Supportive care: comprehensive conservative care in end‐stage kidney disease. Clin J Am Soc Nephrol. 2016;11(10):1909‐1914. 27510453 10.2215/CJN.04840516 PMC5053791

[33] Fox S , Chesla C . Living with chronic illness: a phenomenological study of the health effects of the patient–provider relationship. J Am Acad Nurse Pract. 2008;20(3):109‐117. 18336687 10.1111/j.1745-7599.2007.00295.x

[34] Frantsve L , Kerns R . Patient‐provider interactions in the management of chronic pain: current findings within the context of shared medical decision making: table 1. Pain Medicine. 2007;8(1):25‐35. 17244101 10.1111/j.1526-4637.2007.00250.x

[35] Meredith L , Orlando M , Humphrey N , Camp P , Sherbourne C . Are better ratings of the patient‐provider relationship associated with higher quality care for depression? Med Care. 2001;39(4):349‐360. 11329522 10.1097/00005650-200104000-00006

[36] Pilnick A , Dingwall R . On the remarkable persistence of asymmetry in doctor/patient interaction: a critical review. Soc Sci Med. 2011;72(8):1374‐1382. 21454003 10.1016/j.socscimed.2011.02.033

[37] Tsai D . How should doctors approach patients? A Confucian reflection on personhood. J Med Ethics. 2001;27(1):44‐50. 11233378 10.1136/jme.27.1.44 PMC1733353

[38] Hussain J , Flemming K , Murtagh F , Johnson M . Patient and health care professional decision‐making to commence and withdraw from renal dialysis: a systematic review of qualitative research. Clin J Am Soc Nephrol. 2015;10(7):1201‐1215. 25943310 10.2215/CJN.11091114 PMC4491298

[39] Stacey D , Légaré F , Lewis K , et al. Decision aids for people facing health treatment or screening decisions. Cochrane Database Syst Rev. 2017;4:CD001431. 28402085 10.1002/14651858.CD001431.pub5 PMC6478132

[40] Agoritsas T , Heen AF , Brandt L , et al. Decision aids that really promote shared decision making: the pace quickens. BMJ. 2015;350(feb10 14):g7624. 25670178 10.1136/bmj.g7624 PMC4707568

[41] Robinski M , Mau W , Wienke A , Girndt M . Shared decision‐making in chronic kidney disease: a retrospection of recently initiated dialysis patients in Germany. Patient Educ Couns. 2016;99(4):562‐570. 10.1016/j.pec.2015.10.014. 26527307

[42] Ho A . Relational autonomy or undue pressure? Family's role in medical decision‐making. Scand J Caring Sci. 2008;22(1):128‐135. 18269432 10.1111/j.1471-6712.2007.00561.x

[43] Blackler L . Compromised autonomy: when families pressure patients to change their wishes. J Hospice Palliative Nursing. 2016;18(4):184‐191.

[44] Krishna L . Best interests determination within the Singapore context. Nursing Ethics. 2012;19(6):787‐799. 22547489 10.1177/0969733011433316

[45] Strategy Group Singapore . Population in brief 2018. Available at https://www.strategygroup.gov.sg/docs/default-source/default-document-library/population-in-brief-2018.pdf. Accessed December 11, 2018.

[46] Calnan M , Almond S , Smith N . Ageing and public satisfaction with the health service: an analysis of recent trends. Soc Sci Med. 2003;57(4):757‐762. 12821022 10.1016/s0277-9536(03)00128-x

