# Supplementary material for: Perspectives on decision making amongst older people with end‐stage renal disease and caregivers in Singapore: A qualitative study
Source: Health Expect. 2019 Aug 16;22(5):1100–10. doi: 10.1111/hex.12943 (PMC6803400; doi:10.1111/hex.12943)
Supplement: Supplementary file 1 [file HEX-22-1100-s001.docx]

**Appendix 1. Interview Guide**

Patient Interview Guide

| **Introductory Questions:** |
| --- |
| To start, could you tell us a bit about your family?  Can you tell me a bit about your living situation? Do you live with anyone? Who?  Who helps you with your daily tasks like chores or cooking? |
| **Medical History:** |
| Thinking back, can you tell me about your health history?  Can you tell me about how you were diagnosed with weak kidneys/kidney disease?  What do you think contributed to your weak kidneys/kidney failure? |
| **Medication & Dialysis:** |
| Are you currently undergoing Dialysis?  What medications do you currently take?  Does anyone help you manage or take your medications?  Do you experience any difficulties in taking your medication?  Now thinking about dialysis, what type of dialysis do you do? |
| **(1) Peritoneal dialysis** |
| How often do you do dialysis?  Where do you do dialysis?  Can you describe to me a typical day when you get dialysis?  Does anyone assist you in doing dialysis?  What problems do you face in doing dialysis?  Who helps you when you face these problems?  Are you experiencing any side-effects from your dialysis?  What about cost – How do you pay for your dialysis every month?  How about all your medical expenses? How do you pay for them?  Since beginning dialysis, what are the changes you have had to make in daily living?  How has your life changed since you started doing dialysis?  Before you started dialysis, were you aware of how your life would be on dialysis?  What were your expectations of dialysis?  Thinking back on the decisions you have made about your treatment, is there anything you might have done differently given what you now know?  Have you ever considered switching from peritoneal dialysis to hemodialysis or non-dialysis management?  What could make the experience of doing dialysis better for you? |
| **(2) Hemodialysis** |
| How often do you go for dialysis per week?  Where do you go for dialysis?  Can you describe to me a typical day for you when you get dialysis?  Could you describe to me a typical day for you when you are not getting dialysis?  What problems do you face in going for dialysis?  Are you experiencing any side-effects from your dialysis?  What about cost – How do you pay for your dialysis every month?  How about all your medical expenses? How do you pay for them?  Since beginning dialysis, what are the changes you have had to make in daily living?  How has your life changed since you started doing dialysis?  Before you started dialysis, were you aware of how your life will be with dialysis?  What were your expectations of dialysis?  Thinking back on the decisions you have made about your treatment, is there anything you might have done differently given what you now know?  Have you considered switching from hemodialysis to peritoneal dialysis or non-dialysis management?  What could make the experience of going for dialysis better for you? |
| **(3) Not on Dialysis** |
| Can you describe to me a typical day for you?  What problems do you face in following your treatment and taking medications?  Does anyone assist you in following your treatment and taking medications?  How do you pay for your medical expenses?  Are you experiencing any side-effects from your medications?  Since you had kidney failure/weak kidneys, what are the changes you have had to make in daily living?  How has your life changed since you had kidney failure/weak kidneys?  Were you aware of how your life will be without dialysis?  What were your expectations of not being on dialysis?  Thinking back on the decisions you have made about your treatment, is there anything you might have done differently given what you now know?  Have you ever considered giving up on non-dialysis management and starting dialysis?  What could be done to improve your current situation? |
| **Healthcare Provider** |
| Moving on, can you tell me about your kidney doctor(s)?  How often do you see your kidney doctor?  Overall, what is your relationship with your kidney doctor like?  Do you talk to any other health care workers for your dialysis or kidney disease? Nurses? Counselors? Can you tell me about your relationship with them?  Could anything be done to improve your relationship with your health care team? OR What could be done for the health care team to understand you? |
| **Dialysis Decision-making – Not on Dialysis** |
| Did your kidney Doctor ever recommend dialysis to you?  Why did you choose not to start dialysis treatment?  Was your family involved when you decided not to start dialysis?  Do you think you will choose dialysis treatment in the future, if prompted by your doctor?  Did you receive counseling before on dialysis or other treatment options for your kidney failure/weak kidneys?  What was the counseling like? |
| **Dialysis Decision-making – On Dialysis** |
| Thinking about dialysis, can you describe to me when and how you started dialysis?  Why did you choose to start dialysis?  Who recommended that you start dialysis?  Was your family involved when you decided to start dialysis?  Did you or your family speak to anyone about dialysis that you know of?  Were you given any treatment choices other than dialysis for your kidney failure?  Did you receive counseling before you started dialysis?  What was the counseling like?  Do you think you will ever stop undergoing Dialysis?  Looking back, would you have taken up Dialysis? |
| **ESRD – Awareness and Information Gathering** |
| Thinking back to when you were first diagnosed with kidney failure/weak kidneys – did you know what kidney failure weak kidneys was/were?  Who told you about kidney failure/weak kidneys?  Can you remember what they told you?  Did anyone else tell you about kidney failure/weak kidneys?  Did you look for other information about kidney failure/weak kidneys?  Where do you prefer to get information about your illness? Why?  What would you say is the overall quality of the information you were given?  What could be done to improve the information given to newly diagnosed kidney failure patients/ patients with weak kidneys?  If you were to receive a brochure on different treatment alternatives, what type of information would you like it to provide?  Thinking back, do you think watching a video of patients sharing their experiences of going through dialysis, would help in making your decision? |
| **Closing Questions** |
| Given all we’ve discussed today, if you were to give advice to a person who is recommended to start dialysis very soon what would it be?  Before we end, do you have any final thoughts that you’d like to share?  This brings us to the end of our interview for today. Do you have any final questions? |
