# Supplementary material for: Perspectives on decision making amongst older people with end‐stage renal disease and caregivers in Singapore: A qualitative study
Source: Health Expect. 2019 Aug 16;22(5):1100–10. doi: 10.1111/hex.12943 (PMC6803400; doi:10.1111/hex.12943)
Supplement: Supplementary file 2 [file HEX-22-1100-s002.docx]

**Appendix 2. Consolidated criteria for reporting qualitative studies (COREQ): 32-item checklist**

| **No** | **Item** | **Guide questions/description** |  |
| --- | --- | --- | --- |
| **Domain 1: Research team and reflexivity** | | | |
| **Personal Characteristics** | | | |
| 1. | Interviewer/facilitator | Which author/s conducted the interview or focus group? | JK; EH; RQ; VH; HLQ |
| 2. | Credentials | What were the researcher's credentials? *E.g. PhD, MD* | JK (MSc); EH(MSc); RQ (MSc); VH (MPH); HLQ (PhD) |
| 3. | Occupation | What was their occupation at the time of the study? | JK;EH;RQ;VH: Research Associates  HLQ: A/Prof |
| 4. | Gender | Was the researcher male or female? | JK: Male; EH;RQ;VH;HLQ: Female |
| 5. | Experience and training | What experience or training did the researcher have? | Qualitative research training and experience – in-depth interviews, coding and analysis |
| **Relationship with participants** | | | |
| 6. | Relationship established | Was a relationship established prior to study commencement? | No |
| 7. | Participant knowledge of the interviewer | What did the participants know about the researcher? e*.g. personal goals, reasons for doing the research* | Reasons for doing the research – to make a decision-making aid |
| 8. | Interviewer characteristics | What characteristics were reported about the interviewer/facilitator? e.g. *Bias, assumptions, reasons and interests in the research topic* | Reasons for doing the research – to make a decision-making aid |
| **Domain 2: study design** | | | |
| **Theoretical framework** | | | |
| 9. | Methodological orientation and Theory | What methodological orientation was stated to underpin the study? *e.g. grounded theory, discourse analysis, ethnography, phenomenology, content analysis* | Combination of inductive and deductive thematic analysis, including the constant comparative method from grounded theory |
| **Participant selection** | | | |
| 10. | Sampling | How were participants selected? *e.g. purposive, convenience, consecutive, snowball* | Purposive |
| 11. | Method of approach | How were participants approached? e*.g. face-to-face, telephone, mail, email* | Face-to-face |
| 12. | Sample size | How many participants were in the study? | 23 |
| 13. | Non-participation | How many people refused to participate or dropped out? Reasons? | 38 people declined or were non-communicative; 1 participant dropped out due to hospitalisation |
| **Setting** | | | |
| 14. | Setting of data collection | Where was the data collected? e*.g. home, clinic, workplace* | Clinic within a general hospital |
| 15. | Presence of non-participants | Was anyone else present besides the participants and researchers? | Yes - other patients, caregivers and staff in the waiting area |
| 16. | Description of sample | What are the important characteristics of the sample? *e.g. demographic data, date* | Reported in methods |
| **Data collection** | | | |
| 17. | Interview guide | Were questions, prompts, guides provided by the authors? Was it pilot tested? | Yes |
| 18. | Repeat interviews | Were repeat interviews carried out? If yes, how many? | No |
| 19. | Audio/visual recording | Did the research use audio or visual recording to collect the data? | Audio recording |
| 20. | Field notes | Were field notes made during and/or after the interview or focus group? | Yes |
| 21. | Duration | What was the duration of the interviews or focus group? | Approximately 30 to 60 minutes |
| 22. | Data saturation | Was data saturation discussed? | Yes |
| 23. | Transcripts returned | Were transcripts returned to participants for comment and/or correction? | No |
| **Domain 3: analysis and findings** | | | |
| **Data analysis** | | | |
| 24. | Number of data coders | How many data coders coded the data? | 2 |
| 25. | Description of the coding tree | Did authors provide a description of the coding tree? | Yes |
| 26. | Derivation of themes | Were themes identified in advance or derived from the data? | Both |
| 27. | Software | What software, if applicable, was used to manage the data? | QSR NVivo 11 |
| 28. | Participant checking | Did participants provide feedback on the findings? | No |
| **Reporting** | | | |
| 29. | Quotations presented | Were participant quotations presented to illustrate the themes / findings? Was each quotation identified? e*.g. participant number* | Yes |
| 30. | Data and findings consistent | Was there consistency between the data presented and the findings? | Yes |
| 31. | Clarity of major themes | Were major themes clearly presented in the findings? | Yes |
| 32. | Clarity of minor themes | Is there a description of diverse cases or discussion of minor themes? | Yes |
